# Anti‐coccidial efficacy of *Enteromorpha prolifera* polysaccharide in indigenous chickens of Northwest Ethiopia

**DOI:** 10.1002/vms3.70037

**Published:** 2024-09-17

**Authors:** Bekalu Muluneh, Mengistie Taye, Tadelle Dessie, Dessie Salilew Wondim, Semahegn Yilkal, Fikirtemariam Aregay, Almaz Habtamu, Aschalew Shitu, Halo Yohans, Teketay Wassie, Xin Wu

**Affiliations:** ^1^ Department of Animal Science College of Agriculture and Environmental Sciences, Bahir Dar University Bahir Dar Ethiopia; ^2^ Department of Animal and Range Sciences Wolaita Sodo University, Dawuro Tarcha Campus Sodo Ethiopia; ^3^ Institute of Biotechnology Bahir Dar University Bahir Dar Ethiopia; ^4^ International Livestock Research Institute (ILRI) Addis Ababa Ethiopia; ^5^ Institute of Animal Sciences, Department of Animal Breeding and Husbandry University of Bonn Bonn Germany; ^6^ Agricultural Development Center Bahir Dar University Bahir Dar Ethiopia; ^7^ Bahir Dar Animal Disease Investigation and Diagnostic Laboratory Bahir Dar Ethiopia; ^8^ Department of Veterinary Science College of Agriculture and Environmental Sciences, Bahir Dar University Bahir Dar Ethiopia; ^9^ Department of Molecular Microbiology and Immunology Oregon Health and Science University Portland Oregon USA; ^10^ Key Laboratory of Agro‐Ecological Processes in Subtropical Region, National Engineering Laboratory for Pollution Control and Waste Utilization in Livestock and Poultry Production Chinese Academy of Sciences Changsha China; ^11^ Tianjin Institute of Industrial Biotechnology Chinese Academy of Sciences Tianjin China

**Keywords:** anti‐coccidial, chicken, coccidiosis, efficacy, enteromorpha polysaccharide

## Abstract

**Background:**

A variety of bioactive compounds isolated from various botanical sources have been found to have therapeutic and immunotherapeutic effects on chicken coccidiosis.

**Aim:**

This study aimed to evaluate the anti‐coccidial potential of *Enteromorpha prolifera* polysaccharide (EP) in indigenous chickens in Northwest Ethiopia.

**Materials and Methods:**

A total of 78 male indigenous chickens were used for this study. The study had two treatment groups: (1) the EP non‐supplemented group (those fed on diets without EP and *Eimeria* oocyst inoculated) and (2) the EP group (those receiving diets supplemented with 400 mg EP/kg diet and *Eimeria* oocyst inoculated). Each treatment group had five replications. Following fourteen days of EP supplementation, 1.5 × 10^4^ oocysts of mixed *Eimeria* species were inoculated into individual birds.

**Results:**

EP‐supplemented chicken showed significantly lower (*p* < 0.05) oocyst counts compared to non‐supplemented ones on 9 and 11 days post‐challenge. In addition, chickens in the EP‐supplemented group showed less severe lesion scores, with an average score of 1.33. Chickens that received EP showed a maximum of 27.27% protection against lesions. In contrast, the non‐supplemented chickens had a lower percentage of protection (19.83%). The maximum anti‐coccidial index value (146.98) was obtained from EP‐supplemented chickens. Chickens in the EP‐supplemented group exhibited a significantly higher (*p* < 0.05) weight gain.

**Conclusion:**

Overall, the inclusion of EP in chickens' diets shows promise as a potential anti‐coccidial strategy. However, additional research is required to explore the mechanisms by which EP in chickens’ diet could involve in increasing the protection ability of chickens against coccidiosis.

## Graphical abstract

• *EP* ^+^ chickens showed significantly lower oocyst counts and less severe lesion scores compared to *EP* ^−^ chickens.

• Chickens receiving EP exhibited the maximum protection against lesions and higher weight gain.

• The results suggest that *Enteromorpha* polysaccharide has the potential to be used as an effective anti‐coccidial strategy in chicken diets.

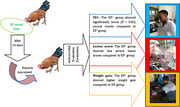

## 1 INTRODUCTION

Avian coccidiosis is an important apicomplexan parasitic disease of poultry birds, having a negative correlation with the bird's ability to reproduce and perform well in terms of growth (Mumtaz et al., 2021). The immune suppression may be brought on in a subclinical form, followed by secondary diseases (Shahid et al., 2020). It is one of the main infectious diseases that affect chicken production efficiency and is spread by *Eimeria* (a type of protozoa), having different species (Chapman, 2014). It has been established that seven host‐specific *Eimeria* species; *E. acervulina*, *E. maxima*, *E. tenella*, *E. brunetti*, *E*. *necatrix*, *E. praecox* and *E. mitis* are responsible for economic losses in chickens (Blake et al., 2020). In every region of the world, coccidiosis results in a significant financial loss for the production of poultry. This parasitic disease could cause an estimated global economic loss of 2.4–3 billion dollars per year (Gordillo Jaramillo et al., 2021; Kadykalo et al., 2018; Rizwan et al., 2022; Zhang et al., 2023). Inadequate ventilation, the presence of sporulated oocysts in the environment for long period, the absence of an all‐in‐all‐out system and additional stressors such as dietary changes and immunosuppressive effects are all potential risk factors for this disease (Adjei‐Mensah & Atuahene, 2023).

Anti‐coccidial drugs and vaccinations are the main contributors to the prevention and control of this issue but with varying degrees of success (Khater et al., 2020). Due to the associated drawbacks of existing control tactics, such as the growth of drug‐resistant strains, the high cost of vaccines and drug residues in meat and eggs, there is also a growing concern about the use of alternative control approaches (Awais et al., 2011). The most popular and widely used technique for the prevention and control of avian coccidiosis is the use of preventive and therapeutic anti‐coccidial medications (Snyder et al., 2021). Various alternative control methods, including probiotics, organic acids, essential oils and antioxidants, are currently under investigation (Kalkal et al., 2021; Raza et al., 2022). However, polysaccharides derived from plants, algae and cereals, such as the polysaccharide extract from *Enteromorpha prolifera*, are garnering increased attention compared to these alternatives. This growing interest stems from their potential for broad effectiveness and non‐specific immunity. Recent studies have highlighted the significance of polysaccharides sourced from diverse plant origins, showcasing their diverse biological functions. These properties include hypolipidemic, hypoglycaemic, antioxidant, anti‐tumour, anti‐viral, anticoagulant, anti‐inflammatory and immunomodulatory effects (Ahmad, 2021; Long et al., 2021; Luo et al., 2021; Zhu et al., 2021). Moreover, these compounds are prevalent and essential biological macromolecules found in plants, recognized for their safety and minimal toxicity (Ahmad, 2021).

*E. prolifera* is a seaweed‐green algae with a long history of use as a traditional medicine and food (Wassie, Niu et al., 2021). One of the primary biologically active components of *E. prolifera* is a sulphated polysaccharide, which is responsible for the immune‐modulating, hypolipidemic, antitumor, anti‐ageing, antibacterial, anticoagulant, antiviral and anticancer properties (Wassie, Niu et al., 2021; Zhang et al., 2022). Previous studies indicated that chickens fed seaweed polysaccharides performed better in terms of growth and production, breast muscle yield, egg quality, antioxidant capacity and intestinal morphology (Guo et al., 2020; Liu et al., 2021; Wassie, Lu et al., 2021; Zhao et al., 2021). Even though several bioactive molecules, such as polysaccharides extracted from various botanical sources, have been discovered for their therapeutic and immunotherapeutic effects on chicken coccidiosis, the anti‐coccidial effects of *E. prolifera* polysaccharide (EP) have not yet been well investigated. Therefore, this study aimed to evaluate the anti‐coccidial potential of EP in chickens.

## 2 MATERIALS AND METHODS

### 2.1 Study location

The experiment was conducted at Zenzelima campus of Bahir Dar University, located in Northwest Ethiopia at 11° 37′ north latitude and 37° 29′ east longitude and an elevation of 1912 m above sea level. The average daily minimum and maximum temperatures were 7 and 29°C, respectively. The average annual rainfall is 1445 mm (Woreda Agriculture Office).

### 2.2 Source of *Enteromorpha* polysaccharide (EP)

The EP, extracted from the marine algae *E. prolifera*, provided by Qingdao Seawin Biotechnology Group Co., Ltd. was used for the experiment. The EP was extracted following a procedure previously reported by Liu et al. (2021). Accordingly, the water‐soluble sulphated polysaccharides of EP were extracted from *E. prolifera* using an enzymatic method. Briefly, the algae were washed in distilled water, dried at 60°C and then minced to create homogenate powder. After the algae powder was soaked in water, it underwent a step‐by‐step enzymatic treatment using pectinase, cellulase and papain at 50°C for 1.5 h. To obtain the polysaccharide products, the enzyme reaction was first inactivated by heating the reaction at 90–100°C for 10 min, followed by an instantaneous cooling on an ice bath, centrifugal concentration, ethanol precipitation and spray drying (Lv et al., 2013). High‐performance liquid chromatography (HPLC), in accordance with the previously reported protocols (Yu et al., 2017), was used to determine the monosaccharide composition. According to the HPLC analytical findings, the EP employed in this study contained the monosaccharides rhamnose (Rha), glucuronic acid (GlcA), xylose (Xyl), glucose (Glc) and galactose (Gal), with the molar percentages being 40.6%, 38.2%, 9.3%, 5.6% and 6.3%, respectively.

### 2.3 Preparation of parasite for infection

Faecal samples were collected from the local chicken suspected of being naturally infected with coccidiosis around Bahir Dar, Ethiopia. The samples were checked for the presence of coccidial eggs (oocysts) using the floatation technique, following the procedure used by Wondimu et al. (2019). The absence of other protozoal oocysts and helminth eggs was checked during various stages of examination, such as direct faecal smear and flotation techniques, sporulation assessment and oocyst dose estimation using the McMaster egg counting slide. To ensure the uniform concentration of *Eimeria* oocysts in all chicken droppings, we thoroughly mixed all positive samples using a pestle and mortar, adding 2.5% potassium dichromate (K_2_Cr_2_O_7_) for sporulation using the standard guidelines provided by Ryley et al. (1976). The sporulation of oocysts was confirmed with direct microscopic examination. The number of oocysts per gram of droppings was estimated using the McMaster slide. Sporulated oocysts were washed with phosphate‐buffered saline (PBS) and used in the challenge experiment by adjusting the dose to 1.5 × 10^4^ with PBS following previous studies by Reid (1970) and Chapman (2002). Each challenge dose was taken with shaking to prevent variations in the number of oocysts in the suspension.

### 2.4 Experimental design and chicken management

A total of 78 adult indigenous male chickens (22 weeks of age) were used in the study. Until they reached this age, they were kept in an intensive management system and received vaccinations for the major diseases (Marek's disease, Newcastle disease, Gumboro, Fowl Typhoid and Fowl Pox) as per the recommended vaccination schedule, and they also received treatment as required. The chickens were fed a commercial starter ration (up to Week 8) and a grower ration (from Week 9 to 20), which were obtained from Alema Koudijs Feed PLC., and they were given clean and fresh water ad libitum. Prior to allocation to each pen, the body weights of all chickens were measured and then chickens were randomly assigned into 2 treatment groups consisting of 39 chickens each, with 5 replications using a Completely Randomized Design. The body weight of the chickens in each treatment group was kept similar. Eight chickens were allocated to each pen, except for two pens that contained seven chickens each. The first group (Control group) was fed a commercial ration, and the second group received a commercial ration supplemented with 400 mg EP/kg diet (EP group), according to the recommended dose (Guo et al., 2020), for 14 consecutive days. The amount of feed was adjusted weekly by considering the developmental stages of the chickens (National Research Council, 1994). Chickens were checked for coccidiosis before the start of the experiment, and after 14 days of the start of the experiment, the EP‐supplemented and non‐supplemented groups were infected with 1.5 × 10^4^ oocysts of mixed *Eimeria*, including *E. acervulina, E. necatrix, E. maxima* and *E. tenella* per bird with PBS. The body weight of chickens was recorded before inoculation and on the 12th day post‐inoculation (the final body weight [FBW]). As egg shedding was expected after 4 days (Mesa‐Pineda et al., 2021), a faecal (dropping oocyst) count was taken on Days 5, 7, 9, 11 and 12 post‐inoculation. A total of 24 chickens (12 from the EP‐supplemented group and 12 from the non‐supplemented group) were slaughtered for small intestine and ceca lesion evaluation.

### 2.5 Data collection

The anti‐coccidial efficacy of EP was assessed based on body weight gain, feed conversion ratio (FCR), survival rate, oocyst value, lesion score, per cent protection against lesions and the anti‐coccidial index (ACI). The initial body weights (IBW) and FBW) were used to calculate the average daily gain (ADG):ADG=(FBW−IBW)/(no.ofexperimentaldays). Feed offered and feed leftovers were measured daily using a sensitive balance to calculate the average daily feed intake (ADFI) and FCR. FCR was calculated as ADFI/ADG. The survival rate was estimated by dividing the number of survived chickens by the initial number of chickens. The relative weight gain rate was determined by dividing the weight gains by IBW. The oocyst value was calculated using the formula described previously (Shah et al., 2010). The per cent protection against lesions was calculated using the formula, Protectionagainstlesions%=(IUG−ITG/IUG)×100; where IUG is infected untreated/EP non‐supplemented group; ITG is the infected treated/EP‐supplemented group (Khaliq et al., 2017). Lesion scores of the chickens from both groups were calculated by the method of Reid (1970); 0 for healthy or showing no signs of infection; 1 for mild lesions; 2 for moderate lesions; 3 for severe lesions; and 4 for extremely severe lesions.

ACI was calculated based on the formula described by Zhi‐Qiang et al. (2022):

$$\begin{array}{ccc} \;ACI & = & \left(\mathrm{relative}\;\mathrm{weight}\;\mathrm{gain}\;\mathrm{rate}+\mathrm{survival}\;\mathrm{rate}\right)\\ & & -\;(\mathrm{lesion}\;\mathrm{rate}+\mathrm{oocyst}\;\mathrm{rate}). \end{array}$$

### 2.6 Statistical analysis

The collected data were analysed by using R 4.1.3 software through the Mann–Whitney *U* test. Origin software was used to construct graphs. Data presented as a percentage, index value and mean value were considered statistically significant when *p *< 0.05. The model used for statistical analysis was as follows:

$$Y_{ij}=\mu +F_{i}+e_{ij}$$

where *Y_ij_* is the response variable; *μ* is the overall mean; *F_i_* is the fixed effect of feed type (*I* = EP‐supplemented and non‐supplemented); and *e_ij_* is the effect of random error.

## 3 RESULTS

### 3.1 Oocyst count

**FIGURE 1:**
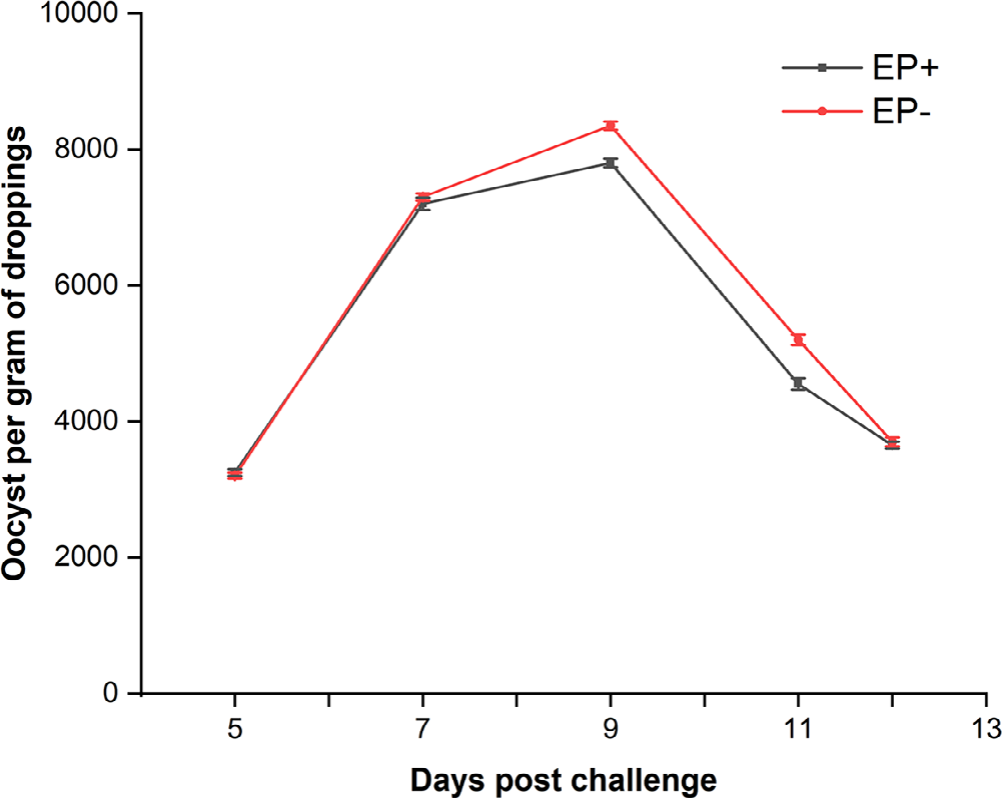
Oocyst per gram of droppings in *Enteromorpha prolifera* polysaccharide (EP)–supplemented (EP^+^) and non‐supplemented (EP^−^) chicken groups challenged with *Eimeria* species.

The EP‐administered chickens showed significantly lower (*p* < 0.05) oocyst counts as compared to non‐supplemented chickens on days 9 and 11 post‐challenge (Figure 1).

### 3.2 Lesion scores and protection against lesions

Chickens from the EP‐supplemented group had significantly lower (*p* < 0.05) severe lesion score (1.33) than non‐supplemented ones, which showed a relatively higher lesion score (1.83). A significant (*p* < 0.05) higher per cent protection against lesions (27.27%) was observed in chickens supplemented with EP than non‐supplemented chickens (Table 1).

**TABLE 1:** The lesion scoring and per cent protection against lesions for *Enteromorpha prolifera* polysaccharide (EP) supplemented (EP^+^) and non‐supplemented (EP^−^) chicken groups.

Treatment groups	Total lesion scoring of chickens					Protection against lesions (%)
	0	1	2	3	4	
EP^+^	0	8	4	0	0	27.27^a^
EP^−^	0	2	10	0	0	19.83^b^

*Note*: Means across a row with different superscript letters (a and b) denote significant differences at *p* < 0.05; a lesion score from 0 (no lesion) to 4 (extremely sever lesion) was given based on Reid (1970).

### 3.3 Anti‐coccidial indices

Relative weight gain rate, survival rate, lesion value and oocyst value were determined to calculate the ACI (Table 2). A relatively higher anti‐coccidial index value (146.98) was obtained from EP‐supplemented chickens than from EP‐non‐supplemented chickens.

**TABLE 2 A:** nti‐coccidial evaluation parameters used for calculation of anti‐coccidial index (ACI) in *Enteromorpha prolifera polysaccharide* (EP)‐supplemented and non‐supplemented indigenous chickens of Northwest Ethiopia.

Parameters	Treatment groups	
	EP^+^	EP^−^
Relative weight gain rate (%)	52.99^a^	25.00^b^
Survival rate (%)	100	100
Lesion value	1.33^b^	1.83^a^
Oocyst value	4.68	4.91
Anti‐coccidial index	146.98	118.26

*Note*: Means across a row with different superscript letters (a and b) denote significant differences at *p* < 0.05.

### 3.4 Post‐challenge average daily gain and feed conversion ratio

**FIGURE 2:**
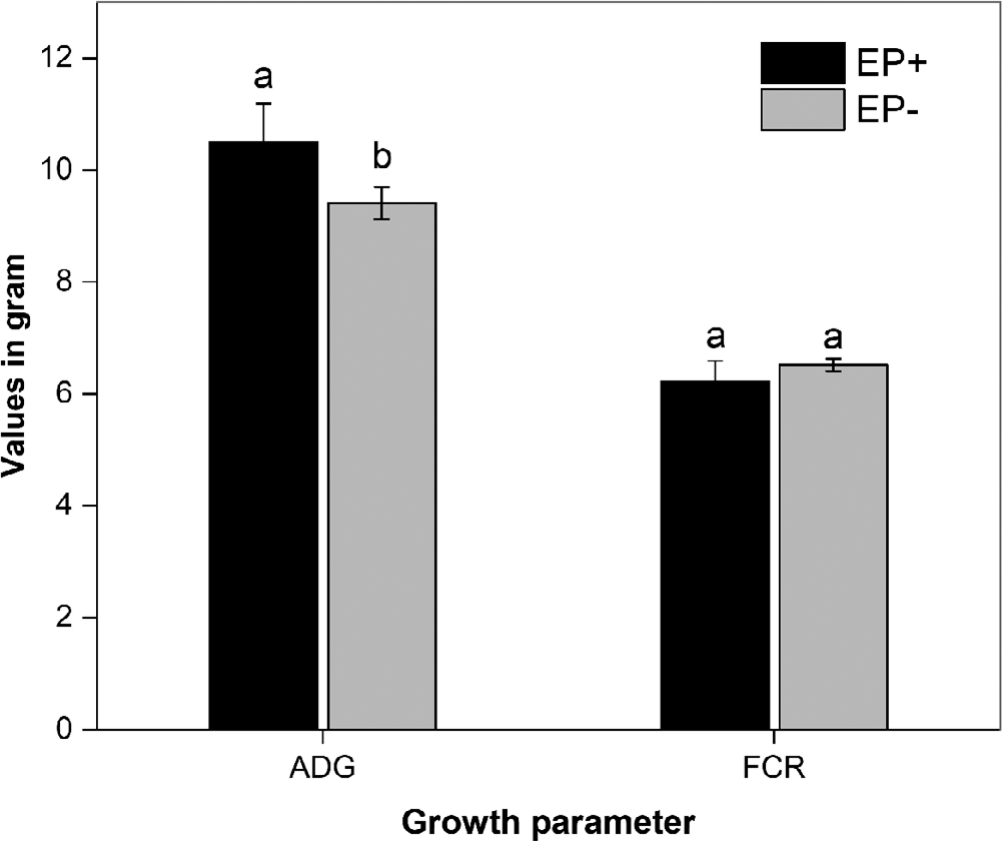
The average daily gain (ADG) and feed conversion ratio (FCR) of *Enteromorpha prolifera* polysaccharide (EP)–supplemented (EP^+^) and non‐supplemented (EP^−^) chicken groups during the coccidiosis challenge.

The average daily weight gains and FCR of EP‐supplemented and non‐supplemented chickens during the coccidiosis challenge were calculated (Figure 2). The weight gain difference between chickens in the EP^+^ and EP^−^ group was statistically significant (*p* < 0.05) but EP supplementation had no significant effect on the FCR of chickens.

## 4 DISCUSSION

Previous findings demonstrated that sulphated polysaccharides from marine algae elicit a variety of biological actions, including immune modulation, antioxidant, anti‐diabetic and hypolipidemic effects, in addition to its usage as food and traditional medicine (Wassie, Lu et al., 2021).

In the current study, the chickens fed EP had relatively reduced oocyst counts. Similar results were obtained by another study that focused on various plant‐derived polysaccharides and herbs (Ali et al., 2019; Ullah et al., 2014; Zhang et al., 2020). The activation of antioxidant enzymes, such as superoxide dismutase and catalase by EP, may have an anti‐sporulation impact by interfering with the parasite life cycle, such as blocking oxygen access and inhibiting certain sporulation‐related enzymes. The Toll‐like receptor‐4 (TLR‐4) and NF‐B signalling pathways are activated by EP supplementation, which prompts an immunological response (Zhang et al., 2022). The reduced oocyst count in the chickens receiving EP supplements might be related to the improvement of cytokines (IL‐2, IL‐4 and IFN‐γ) mRNA expression and TLR‐4 genes, which increase mucosal immunity. In another study, Zhi‐Qiang et al. (2022) reported that IL‐2, IFN‐γ and IL‐17 cytokines play important roles in resistance to coccidiosis infection.

The chickens fed EP had fewer intestinal lesions compared to the EP non‐supplemented chickens. Similar conclusions were made by Ullah et al. (2014) and Amerah and Ravindran (2015), who reported that chickens fed different plant‐derived polysaccharides and compounds exhibit lower lesion scores than control ones. According to Khaliq et al. (2017), the effects of polysaccharides on the microbes of the intestinal tract, reduced bowel putrefaction that subsided or decreased inflammation or the lining of the intestine layer with aloe biomolecules could all be contributing factors to these less severe intestinal lesions. In a previous investigation, mucin‐2 and occluding‐1 mRNA expressions were shown to be increased in the ileum and jejunum of chickens receiving EP supplements (Wassie, Niu et al., 2021). This phenomenon could decrease the intestinal lesion in EP‐consuming chickens as the mucus secreted by mucin‐2 protects the epithelial cells from pathogenic microbes. Dietary supplementation of plant‐derived polysaccharides increases the serum concentration of IgA (Zhang et al., 2022). This IgA can lessen the number of bacteria by binding them to the designated antigens, limit their access to the mucosal membrane of the intestine and also lessen the severity of lesions (Kulkarni et al., 2010).

A higher per cent protection against lesions was observed on chickens supplemented with EP than non‐supplemented chickens. Previous studies also reported that *Aloe vera* polysaccharides increased intestinal macrophage and T‐lymphocyte activity to prevent the penetration of *Eimeria* species, leading to higher protection rates and lower lesion scores in infected birds (Khaliq et al., 2017). EP supplementation could increase caecal short‐chain fatty acids (SCFAs) content, especially acetate, butyrate and propionate, indicating that EP improved caecal metabolites (Wassie, Niu et al., 2021). The SCFAs, particularly butyrate and propionate, provide energy for the immune cell by triggering intestinal gluconeogenesis and enhancing the synthesis of inflammatory and effector cytokines and antigen presentation (Shi et al., 2011). The relatively higher per cent protection against lesions in chickens supplemented with EP might be due to the enhancement of SCFAs in the gut that contributes to intestinal immune response and gut barrier function (Yu et al., 2004).

The ACI reflects the comprehensive ability of any compound against coccidial infection. According to Zhi‐Qiang et al. (2022), values lower than 120 depict that the compound or drug has no anti‐coccidial activity, whereas a medium effect ACI is between 120 and 160; a good effect ACI is between 160 and 180; and an excellent effect ACI is more than 180. In the present study, *EP*‐administered chickens exhibited an ACI value of 146.98, so EP can be considered a partially effective therapeutic/immunotherapeutic regime against coccidiosis. An ACI value of 118.26 was also recorded for EP^−^ chicken group, which could be due to the self‐limiting nature of the *Eimeria* infection in chicken due to genetic and environmental factors, such as host age and infective dose.

In the current study, the EP group exhibited relatively a higher average daily weight gain post‐challenge. Similarly, Awais et al. (2018) reported higher daily weight gains in chickens administered with polysaccharides from sugarcane bagasse. Improvement of intestinal health in EP‐administered groups might be the reason for their relatively higher weight gain. The non‐significant difference in FCR among treatment groups might be due to the effect of other environmental factors, such as feed intake and nutrient utilization.

## 5 CONCLUSION

The results of this study suggest that incorporating EP into the diet of chickens may serve as an effective anti‐coccidial strategy. Therefore, the current findings provide new insights for using EP as an immune stimulant in chickens. However, further investigations focusing on microbiota profiles are necessary to elucidate the underlying mechanism of action.

## AUTHOR CONTRIBUTIONS

**Bekalu Muluneh**: Conceptualization; data curation; formal analysis; funding acquisition; investigation; methodology; resources; software; validation; visualization; writing – original draft; writing – review and editing. **Mengistie Taye**: Conceptualization; data curation; funding acquisition; investigation; methodology; project administration; resources; supervision; validation; writing – original draft; writing – review and editing. **Tadelle Dessie**: Methodology; supervision; validation; writing – original draft; writing – review and editing. **Dessie Salilew Wondim**: Methodology; supervision; writing – original draft; writing – review and editing. **Semahegn Yilkal and Xin Wu**: Resources; writing – original draft; writing – review and editing. **Fikirtemariam Aregay and Almaz Habtamu**: Investigation; resources; writing – original draft; writing – review and editing. **Aschalew Shitu and Halo Yohans**: Investigation; methodology; resources; writing – original draft; writing – review and editing. **Teketay Wassie**: Data curation; methodology; resources; validation; visualization; writing – original draft; writing – review and editing.

## CONFLICT OF INTEREST STATEMENT

The authors have no conflicts of interest.

## FUNDING INFORMATION

Institute of Biotechnology of Bahir Dar University and Wolaita Sodo University

## ETHICS STATEMENT

This study was reviewed and approved by Bahir Dar University College of Agriculture and Environmental Science (BDU‐CAES), Ethiopia.

### PEER REVIEW

The peer review history for this article is available at https://www.webofscience.com/api/gateway/wos/peer‐review/10.1002/vms3.70037.

## Data Availability

Data are available on request from the author.
